# Durability performance of concrete incorporating carbonated recycled coarse aggregates: a review

**DOI:** 10.1038/s44296-025-00071-x

**Published:** 2025-08-18

**Authors:** Zhidong Zhang, Ueli Angst, Viacheslav Troian, Bingbing Guo, Qiang Zeng

**Affiliations:** 1 Institute for Building Materials, ETH Zurich, Zurich, Switzerland; 2 Department of Technology of Building Structures and Products, Kyiv National University of Construction and Architecture, Kyiv, Ukraine; 3 School of Civil Engineering, Xi’an University of Architecture and Technology, Xi’an, China; 4 Department of Civil Engineering and Architecture, Zhejiang University, Hangzhou, China

**Keywords:** Civil engineering, Structural materials

## Abstract

Carbonation of recycled coarse aggregates (RAs) has drawn significant attention in recent years. This study shows that most durability indicators of carbonated RAs concrete (cRAC) are lower than natural aggregate concrete, while compared to concrete with non-carbonated RAs, all durability indicators of cRAC are improved by 13–55% because of the densified microstructure after carbonation. Optimized carbonation methods (i.e., adjusting moisture content and using biomaterials) are suggested to improve the quality of carbonated RAs further.

## Introduction

Concrete is the most used man-made material in the world. The use of concrete has two significant environmental impacts. First, the cement manufacturing generates 5–8% man-made CO_2_ each year globally^1^. To reduce carbon footprint in the construction sector, researchers and engineers have tried various ways to reduce CO_2_ emissions during cement production, such as fuel switching, improved energy efficiency, and clinker substitution^2,3^. However, to reach net-zero for cement production, CO_2_ capture, storage, and utilization are unavoidable^1^. Second, an increasing amount of construction and demolition wastes (CDW) due to the accelerated urbanization and industrialization also results in increasing negative environmental impacts^4^. It is estimated that China generated around 1857 million tons of CDW in 2023 and will reach 2029 million tons in 2026^5,6^. The European Commission reported that about 450–500 million tons of CDW are generated every year in Europe^7^, of which at least one third is demolished concrete. According to the United States Environmental Protection Agency’s report^8^, about 600 million tons of CDW, with two-third of demolished concrete, were generated in 2018 in the US. However, the usage of demolished concrete generally follows the linear economic model, meaning that a large portion of CDW is not reused to produce new concrete, but instead used for landfills or road subbases. The recycling rates of CDW vary with the conditions in different countries. About 73% of CDW in the US were used to produce new manufactured products, recycled coarse concrete aggregates (RAs), etc^8^. The recycling rates of demolished concrete in European countries are generally above 80% (e.g., road subbases, RAs), such as Germany, Belgium, and UK, while only about 5% of CDW in China and other developing countries are recycled^5^.

**Fig. 1:**
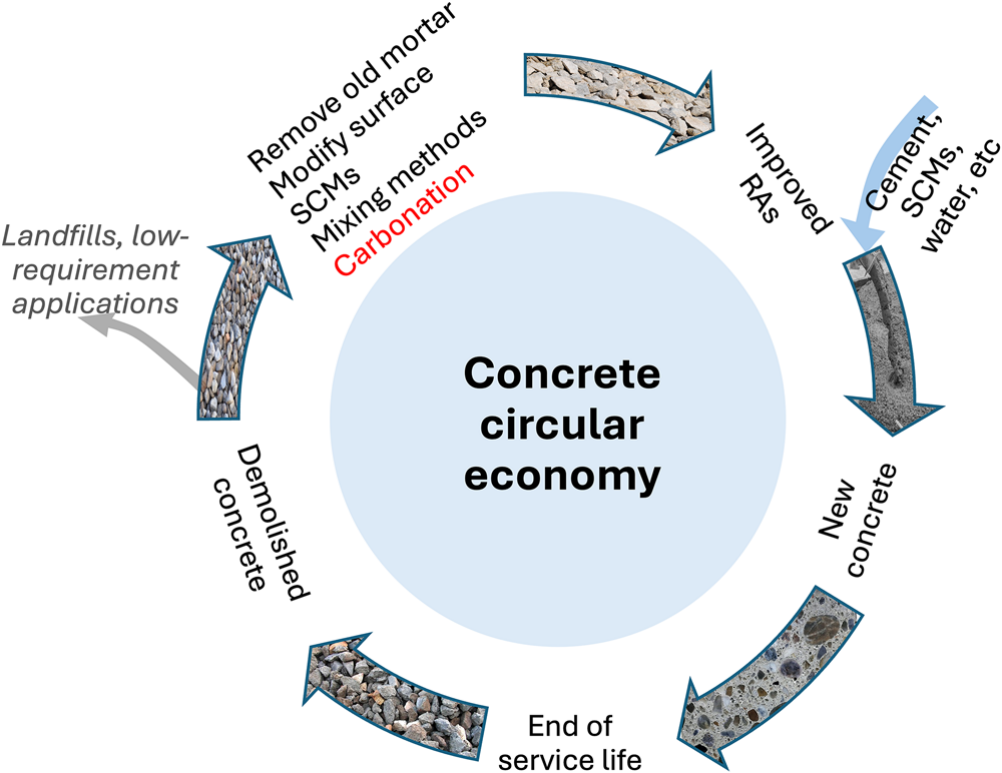
The circular economy model for concrete, which requires to limit the amount of generated waste and low-requirement applications.

To reduce the environmental impacts, the usage of demolished concrete needs to follow the circular economic model as demonstrated in Fig. 1, so that the demolished concrete should be reused to produce new concrete with a very limited amount ending up with landfills (i.e., minimize the gray arrow in Fig. 1). The most widely used way is to crush the demolished concrete and prepare RAs, which can be used to replace a certain portion of natural aggregates (NAs) in newly prepared concrete. Efficient reuse of RAs produced from CDW may help to address the challenges of environmental impacts to large extent, such as preserving natural rock resources, fulfilling the increasing demand for construction materials, reducing CO_2_ emissions due to mining, and saving landfill sites, so the incorporation of RAs in concrete is beneficial for sustainable development of the construction industry. It was estimated that, compared with the exploitation of NAs, the production of RAs could reduce carbon emission by 28–36% depending on the replacement rate of NAs^9,10^. However, RAs have limited structural applications worldwide and generally end up with low-requirement applications, such as road subbases, because of the low aggregate quality compared with NAs. RAs contain up to about 70 wt% of old mortar adhered to the NAs, which leads to worse mechanical properties of RAs than NAs^11,12^. Because of the presence of the adhering mortar, the mesostructure of the RAC is far more complex than that of natural aggregate concrete (NAC)^11,13^. The presence of micro-cracks and residual old mortar increases the porosity of RAs, thus lowering its density and enhancing water absorption. Therefore, the quality of RAs needs to be improved to overcome the low-requirement applications.

Various techniques have been proposed in the past few decades to improve the quality and performance of RAs. Those technical methods belong to different categories, including the removal of residual mortar from RAs, the modification of RAs’ surface, the use of supplementary cementitious materials, the optimization of mixing methods, and carbonation. It was found that removing the amount of adhering mortar in RAs by mechanical methods like rubbing and ball milling can reduce the water absorption rate^14^ and improve the freeze-thaw (FT) resistance^15^. The chemical ways to remove old mortar such as by hydrochloric, sulfuric and acetic acid solutions resulted in the improvement of RAC durability performance^16^. Other methods, including the multi-step concrete mixing, adding fly ash, silica fume and inorganic admixtures, and surface coating of RAs, were reported to enhance the RAC performance^16–19^. However, different chemicals and mechanical treatment methods may result in high CO_2_ emissions, high cost, high energy demand, and other related environmental problems^11,13^.

Recently, the accelerated carbonation treatment of RAs has shown a positive impact on the performance enhancement of RAC^11,13,20,21^. During accelerated carbonation treatment, calcium carbonate is introduced into the pores in RAs. Studies showed that carbonation treatment of OPC-based RAs can increase compressive strength and density, and reduce water absorption and permeability, particularly the interfacial transition zone of carbonated RAs (cRAs) is harder than the non-carbonated RAs^11^. It was reported that concrete prepared with carbonated OPC-based RAs has compressive strength of about 90% of concrete made with NAs^13^. Furthermore, carbonation of RAs is a promising way to reduce the carbon footprint of construction sector. A recent study conducted the industrial scale carbonation of RAs with 15% CO_2_ concentration emitted from a cement plant and showed that CO_2_ uptake is in the order of 2 wt% of demolished concrete^22^. Considering the large quantity of demolished concrete globally, the amount of CO_2_ permanently captured by cRAs is nonignorable^23^. However, the low-value applications of demolished concrete underutilize the CO_2_ storage potential of RAs. Additionally, life cycle assessment showed that the calculated global warming potential for accelerated carbonation treatment is lower than that for the normal two-stage crushing or heating treatments of RAs^24^. Therefore, aiming at reducing the loss of potential CO_2_ capture opportunities and minimizing the environmental impact, the carbonation treatments of RAs are recommended to improve the durability performance of recycled concrete.

This paper provides an overview of the durability performance of recycled concrete with carbonated RAs (cRAC), in particular focusing on well controlled lab experiments. The paper starts with presenting the common carbonation methods and their effects on the properties of RAs, which are then related to the durability indicators of recycled concrete. More insights about carbonation methods, potential durability concerns and research opportunities are then discussed, which may help to tackle the existing problems and further enhance the durability performance of cRAC.

## Carbonation of RAs

### Carbonation methods

This paper only reviews the common carbonation methods that are conducted by research projects in the laboratory, which have well controlled carbonation conditions compared with industrial applications. Generally, RAs are kept in a chamber with a constant CO_2_ concentration, optimized relative humidity (RH), and constant temperature for a certain period. To achieve the fast carbonation, high CO_2_ concentrations are used, from 20 to 100%^11,20,25,26^. It is generally agreed that RH between 50 and 80% is the optimal condition for carbonation^15,27,28^ as there are empty pore spaces for CO_2_ gas diffusion and sufficient water for carbonation reactions. However, for industrial applications, RH and CO_2_ concentration are difficult to be well controlled and maintained constant at the same time in large scale reactors. The emerging technologies such as the atomized water droplet humidity control technology may have a great potential for concrete carbonation but very limited studies can be found in the literature. Room temperature and atmospheric pressure are default for most studies, but with elevated temperature (up to 60 °C) and pressure (between of 10 and 500 kPa), the gas diffusion and carbonation reaction kinetics can be enhanced^11,27,29,30^. Another key variable is the carbonation duration which varies from 1 day to 11 days^27,28,31^, while some studies used much longer carbonation (1 month)^32^. The long durations permit the ingress of CO_2_ deep within an aggregate and convert most calcium hydroxide into calcium carbonate, so the aggregate can be densified^13,33,34^. Nevertheless, the long duration has a risk of over-carbonation of C-S-H, resulting low mechanical strength of the cementitious matrix^35,36^. The supercritical CO_2_ was also used to carbonate RAs and showed enhanced carbonation efficiency^37^, but such a technology requires an autoclave to maintain the high pressure and the critical point of supercritical CO_2_ varies with the moisture condition in the autoclave.

RAs from CDW are partially carbonated during service life and the preparation of RAs, so they contain less alkalis than fresh concrete. Therefore, coating with an alkaline slurry before carbonation was investigated, such as recycled binder paste^38^, lime water^39^, etc. The combined usage of coating and carbonation can significantly enhance properties including mechanical strength and durability of RAC as the additional alkalis induce more calcium carbonate and a denser old mortar. Microbial carbonation by *S. pasteurii* bacteria can also improve the durability performance of RAs^40,41^. However, a limited number of studies have been published on these methods, so this review will only focus on research results of the conventional accelerated carbonation methods using CO_2_ gas.

### Effects of carbonation on microstructural properties of RAs

**Fig. 2:**
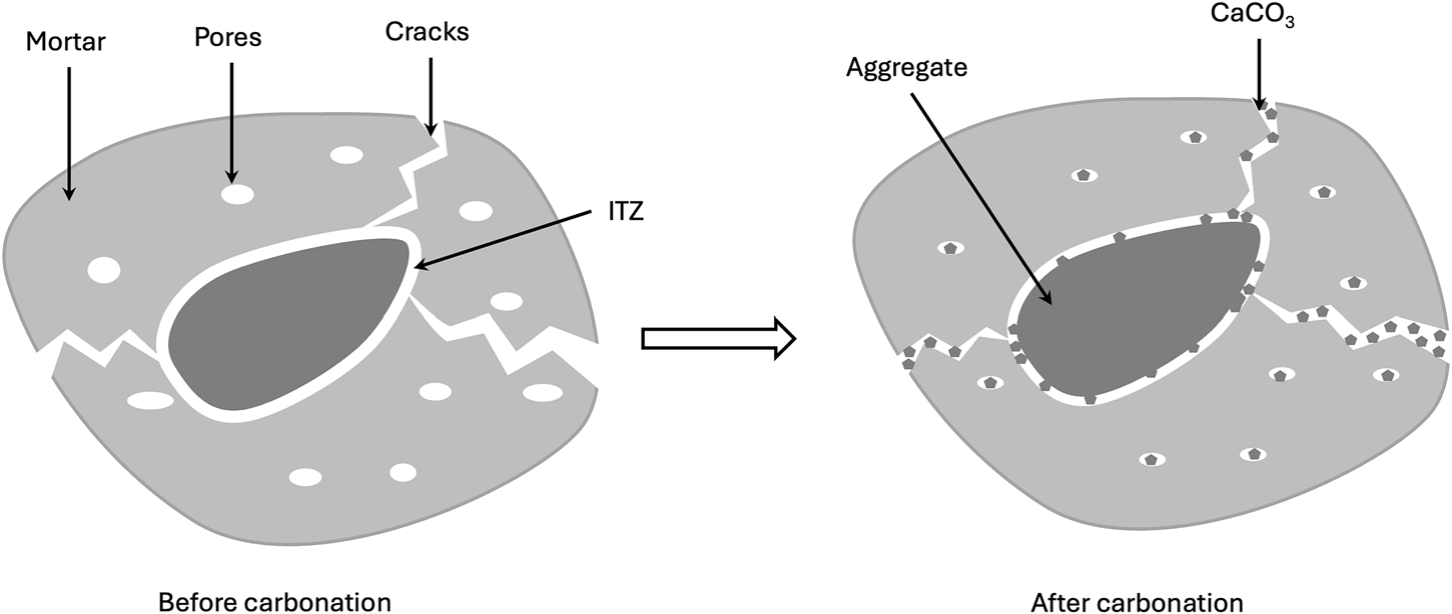
The change of microstructure of an RA after carbonation.

During the production of RAs, crushing and sieving can lead to micro-cracks in the old mortar and further damage interfacial transition zones (ITZs), as shown in Fig. 2. Therefore, there are generally more capillary pathways in RAs than in NAs^42–44^. Enhancement of the microstructure of RAs is one of the great concerns to improve durability properties of RAC^44–47^. The effect of carbonation on the properties of RAs occurs at different locations as CaCO_3_ can precipitate in pores, micro-cracks and ITZs (see the illustration in Fig. 2). Because of more solids formed after carbonation, the macroscopic properties are largely modified, such as the reduction in the measured porosity, and the increase in the density of RAs compared with non-carbonated RAs^26,48^. At the microscopical level, SEM images clearly show that the old mortar becomes denser after carbonation^49^, meaning that the carbonation treatment can not only improve the microstructure of old mortar but also enhance ITZs in RAs due to the precipitation of CaCO_3_. As a result, water absorption of RAs reduces significantly after carbonation, by 20–30%^9,26^ and the micro-hardness of old ITZs is improved^26^.

## Durability of cRAC (recycled concrete with cRAs)

The durability indicators measured for NAC, RAC and cRAC were collected from the literature and compared. Table 1 summarizes the test information performed in the literature, including durability indicators of concrete shrinkage (autogenous and drying), transport properties (water absorption, sorptivity, electrical conductivity, chloride diffusion coefficient), carbonation depth, FT resistance, and alkaline silica reaction (ASR). Different replacement ratios of NAs with (c)RAs can result in different concrete performance. Because the widely used replacement ratio is 100%, this review only focuses on studies with this replacement ratio. As studies used aggregates from various sources with different properties, a direct comparison of the measured values of durability indicators is not possible. Instead, we here compare the relative changes of durability indicators from the same study. The relative change is calculated by

1 $${R}_{x}=\frac{{y}^{x}-{y}_{i}^{x}}{\max \left({y}^{x},{y}_{i}^{x}\right)}$$

where ${R}_{x}$ is the relative change of the durability indicator $x$, and ${y}^{x}$ is the measured value of $x$ for cRAC and ${y}_{i}^{x}$ is the measured value for NAC or RAC depending on which material is compared with cRAC. Following the suggestion by RILEM TC 262-SCI^50^, $\max \left({y}^{x},{y}_{i}^{x}\right)$ is used as the denominator to ensure that negative and positive relative changes are equally scaled. A positive ${R}_{x}$ means that the measured durability indicator for cRAC is higher than NAC or RAC, so the durability of cRAC is impaired and a negative ${R}_{x}$ indicates the enhanced durability.

**Table 1:** Summary of carbonation methods and measured durability indicators in the literature

Publication	Cement type^a^	Concrete mixture	RAs source	RAs type^b^	RAs size	RAs precondition for carbonation	RAs carbonation methods	cRAs pre-wetted?	Measured durability indicators	Tests in detail
Xuan et al. ^11^	-	w/c = 0.55	Prepared and crushed in lab, about 6 months old	Type A	5–10 mm and 10–20 mm	-	100% CO_2_, ~70% RH, 1 and 0.5 bar for 24 h	Lab condition	Drying shrinkage	Length change, 50% RH, until 112 days
									Water absorption	Saturated and oven dry at 105 °C
									Electrical conductivity	ASTM C1760-12, 56 days old
Li et al. ^28^	-	w/c = 0.4, cement 205 kg/m^3^	Self-prepared and crushed, about 6 months old	Type A	5–10 mm and 10–20 mm	-	95% CO_2_, ~70% RH at 20 °C for 3 and 7 days	Pre- soaking for 24 h	Freeze-thaw	300 cycles, 4 h each cycle, between −5 and 20 °C.
Liang et al. ^25^	-	w/c = 0.35, 0.45, and 0.55	Obtained from fresh concrete	Type A	5–20 mm	-	20% CO_2_, ~70% RH at 20 °C	Additional water added	Cl diffusion coefficient	Rapid chloride migration test
									Corrosion rate	36 days immersion in 15% Cl solution, measuring weight loss
Zhang et al. (2015)^20^	P.O. 42.5	w/c = 0.5	Prepared in lab	Type A	0.16–2.5 mm	-	20% CO_2_, ~60% RH at 20 °C	Lab condition	Autogenous shrinkage	ASTM C1698-09, in plastic mold, measuring longitudinal deformation for 3 days
									Drying shrinkage	60% RH and 20 °C, measuring vertical length for 56 days
									Water absorption	Oven dried at 105 °C and capillary saturated
									Cl diffusion coefficient	Rapid chloride migration test
Waseem et al, ^88^	-	w/c = 0.45	Crushed PC samples	Type A	4.75–20 mm	50-7-% RH for 24 h	99% CO_2_ for 2 d	After carbonation	Electrical conductivity	ASTM C1202-12, 28 days old
									Water absorption	ASTM C642-97, 28 days old
									Sorptivity	ASTM C1585-4, 28 days old
Lu et al. ^26^	P.I 42.5	-	Prepared and crushed in lab, about 90 days old	Type A	5–20 mm	-	99.9% CO_2_, 55% RH at 22°C	Pre-wet	Autogenous shrinkage	ASTM 1698-2014
									Drying shrinkage	ASTM C596-09-2017
									Cl diffusion coefficient	ASTM C1556-11
Li et al. ^89^	Type I	w/c = 0.6, cement 450 kg/m^3^	Prepared and crushed in lab, about 180 days old	Type A	4.75–19 mm	50% RH for 1 day	99% CO2, 1 bar, 65% RH for 1 h, 1 day, and 7 days	Additional water added	Sorptivity	ASTM C1585-13
									Electrical conductivity	ASTM C1202–19
Russo and Lollini^32^	CEM II/A-LL	w/c = 0.5	Prepared and crushed in lab, with w/c = 0.46−0.61	Type A	7.5–16 mm	-	100% CO_2_, 65% RH and 20 °C for 1 month	Pre-wet	Water absorption	EN 1097–6
									Sorptivity	EN 13057
									Electrical conductivity	100 mm cubic specimens, measuring resistance between two metal plates
									Cl diffusion coefficient	Rapid chloride migration test
									Carbonation rate	Indoor carbonation at 55–65% RH Accelerated carbonation at 3% CO_2_ and 65% RH
Wang et al. ^56^	Portland cement with fly ash	w/c = 0.48, cement 400 kg/m^3^	From a construction site	Not mentioned, but likely Type A	5–25 mm	-	20% CO_2_, ~70% RH, 20 °C	Additional water added	Drying shrinkage	GB/T 50082-2009, 60% RH and 20 °C, measuring length for 112 days
									Cl diffusion coefficient	GB/T 50082-2009
Abate et al. ^51^	Type 1 OPC	w/b = 0.3	From a local construction waste disposal facility	-	2–12.5 mm	-	5% CO_2_, 50% RH and 20 °C for 7 days	Saturated	Autogenous shrinkage	Sealed, measuring length change at 90 and 113 days
									Drying shrinkage	50% RH, measuring length change at 90 and 113 days
Singh et al. ^52^	OPC	w/c = 0.49, cement 420 kg/m^3^	Prepared and crushed in lab	Type A	4.75–10 mm and 10–20 mm	Ambient dry for 6 h, 40–70% RH	99% CO2, sealed for 2 days	Lab condition	Water absorption	-
									Sorptivity	-
Kazmi et al. ^31^	P.II 52.5R	-	From a concrete recycling facility, total impurities <5%	Type A	<20 mm	50% RH for 7 days	100% CO_2_, sealed for 1 days	Oven dried with extra water before mixing	Water absorption	ASTM C642:2013, oven dried at 105 °C and then immersed in water for 1 days
									Cl diffusion coefficient	Rapid chloride migration test (NT-Build 492-11)
									Carbonation depth	5% CO_2_ and 65% RH for 28 days
Kazmi et al. ^15^	-	w/c = 0.48, cement 138.3 kg/m^3^	From a local concrete recycler, total impurities <5%	Type A	<20 mm	40–70% moisture content	100% CO_2_, 0.8 bar, sealed for 1 days	Oven dried with extra water before mixing	Freeze-thaw resistance	ASTM C666:2015 (Procedure A), 66 cycles
									Sulfate resistance	ASTM C1012:2018, 10% Na_2_SO_4_ solution for 60 days
Grigoletto et al. ^27^	CEM I 52.5 N	c/a = 0.5 and 1.25	From local company, total impurities <5%	Type A	4–20 mm	-	20% CO_2_, 60% RH and 39 °C for 11 days	Pre-saturated until 85% of water absorption	ASR expansion	NFP18-594 standard
Peng et al.^58^,	Type I	w/c = 0.55, cement 350 kg.m^3^	From 50-y old concrete structures	Type A	5–20 mm	50% RH for 3 days	100% CO2 for 1 days	After carbonation	Corrosion rate	At 35 days old, wet/dry cycles (3days/4 days) in 3.5% NaCl solution for 22 weeks
Liang et al. ^57^	-	w/c = 0.5, cement 440 kg/m^2^	Prepared and crushed in lab	Type A	5–20 mm	-	20% CO2, 70% RH for 10 days	After carbonation	Carbonation depth	GB/T 50082-2009, 20% CO_2_, ~70% RH at 20 °C for 56 days
									Freeze-thaw resistance	JTJ 270-1998, temperature −17 to 8 °C, 4 h each cycle, 50 cycles
Li et al. ^90^	CEM I 52.5 N	w/c = 0.6, cement 325 kg/m^3^	From local demolition site	Type A	5–20 mm	50% RH for 1 days	99% CO_2_ for 1 days	Lab condition (50% RH)	Sorptivity	ASTM C1585-13
									Electrical conductivity	ASTM C1202-19, 28 days old

^a^The types of cement in these studies were based on different standards. P.O., P.I. and P.II. are based on China standard GB 175-2007. CEM I and CEM II are based on European standard EN 197-1. Type I is from the American standard ASTM C150.

^b^The types of RAs are classified in European standard EN 206. Type A contains RAs above 90% and RAs + other aggregates above 95%.

### Comparison with natural aggregate concrete (NAC)

**Fig. 3:**
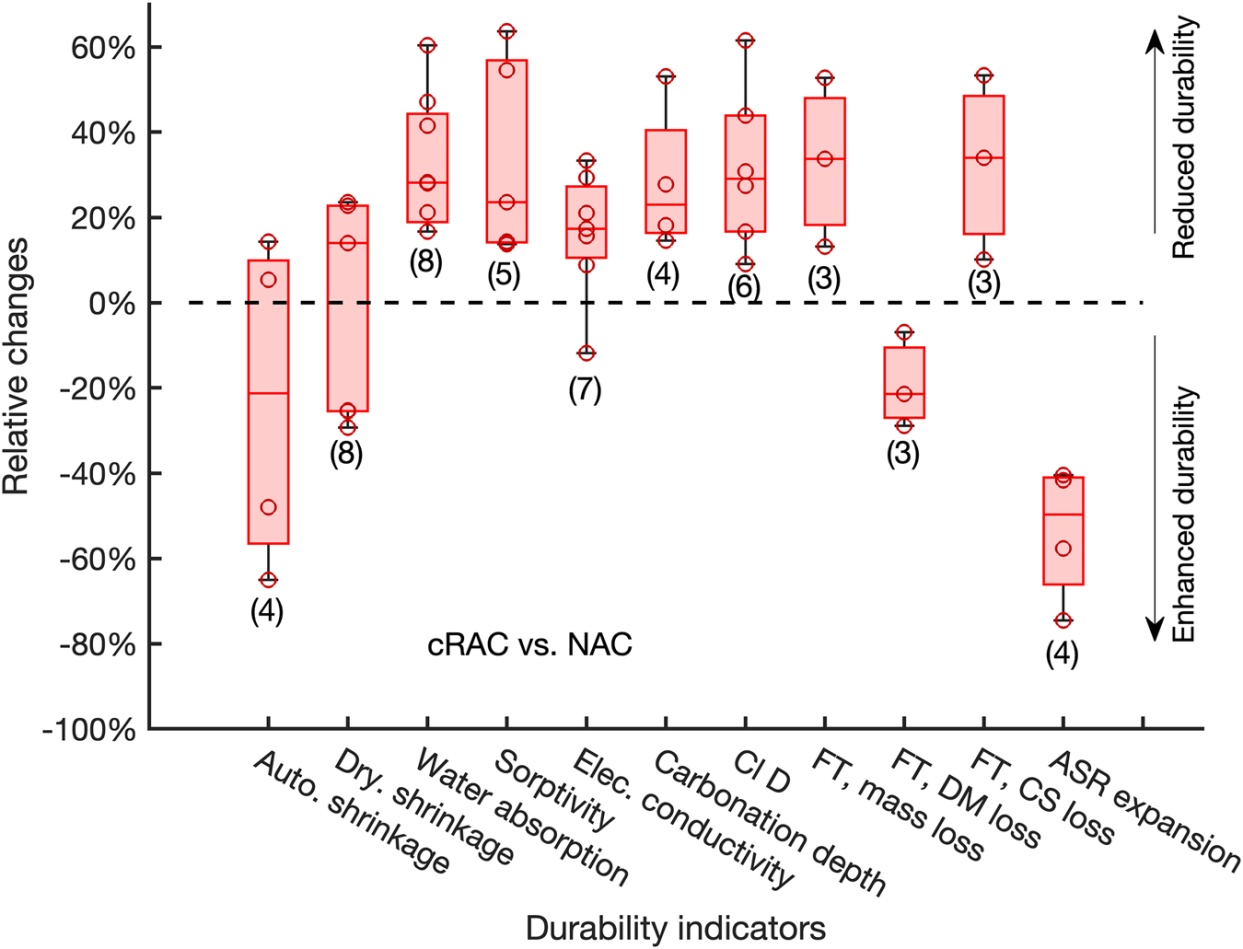
Box plots of relative changes of durability indicators in percentage for cRAC compared with NAC. Each box shows the maximum (top whisker), the 75th percentile (upper half box), the median (middle whisker), the 25th percentile (lower half box) and the minimum (bottom whisker). The parenthesis under the box shows the number of collected data for each indicator. Values of these data points are shown as circles in the plot.

Compared with NAC, the durability indicators of cRAC, including drying shrinkage, water absorption, sorptivity, carbonation depth, chloride diffusion coefficient (Cl D), and ASR expansion, increase as shown in Fig. 3, indicating the reduction of durability. Contradictory results (both reduction and increase) for the measured autogenous and drying shrinkage were found in the literature as shown by the box plot in which the measured data cluster at two ends of the box. The main cause of this contradiction might be the different initial moisture content. After pre-soaking cRAs with water, the measured drying shrinkage was reduced by 25–30% and autogenous shrinkage was reduced by 48–65% (data at the low end of the box in Fig. 3) because cRAs with a high-water content can enhance the internal curing and thus reduce both drying and autogenous shrinkage^51^. Without pre-soaking, increases of 14–31% in drying shrinkage and 5–14% in autogenous shrinkage (data at the high end of the box in Fig. 3) were reported^11,20,26^ which can be explained by the fact that cRAs absorb water during concrete mixing, leaving less water for cement hydration. After concrete hardening, the measured water absorption for cRAC was still higher than for NAC as reported in many studies^11,20,31,32,52^. Water absorption measures the pore volume that can be filled with water under the capillary forces. An average of 30% increase means that capillary pore volume in cRAC is about one third higher than that in NAC. Consequently, sorptivity of the first stage of water uptake, standing for the speed of water penetration into a porous material under capillary pressure^53^, was accelerated as well^32,52^. The increased capillary pore volume may be mainly attributed to the porous old mortar and the ITZs in cRAs.

In line with moisture transport, increases were also reported for electrical conductivity, chloride diffusion coefficient, and carbonation rate. The measured electrical conductivity of cRAC increased by 15–21% for different carbonation schemes compared to NAC^11^, but a reduction was also reported^32^. The electrical conductivity depends on not only the microstructure but also the ionic strength. After 1-month carbonation^32^, the ionic strength in cRAs is lower than the short-carbonated aggregates^11^. Chloride diffusion coefficient was found to increase by 31% in average. Similarly, the longer carbonation of RAs had about 10% increase in chloride diffusion coefficient^32^, much lower than other studies^20,31,52^. As the water-accessible porosity is higher for cRAC than NAC, CO_2_ diffusion is also faster, resulting in fast carbonation of concrete^22^. The carbonation rate increased by 28% in average with a higher increase for the short-term carbonation (28 days)^31^ and a lower increase for the long-term carbonation (~1 year)^32^. The highest increase in transport properties was found for the measured argon gas permeability which has an increase of 200%^11^. It is well recognized that gas permeability is higher than water permeability because gasses have weaker interactions with hydration products than water^54,55^. The increase in transport properties indicates the coarser pore structure in cRAC than NAC.

The FT resistance of concrete is commonly quantified by mass loss, loss of relative dynamic modulus (FT, DM loss) and compressive strength (FT, CS loss) after a certain number of FT cycles^15,28^. It was found that the damage process caused by FT cycles occurred in two stages. In the beginning, the FT cycles accelerated water penetration and the inner cracks in cRAs further enhanced this process, resulting in very minor mass loss. With the increase in water content, the frost damage developed slowly, and the FT mass loss increased. With the increase in the number of cycles, a linearly increasing stage occurs with the spalling of the surface mortar when the FT expansion stress exceeds the tensile strength of concrete. Therefore, the use of cRAs has a small effect on the FT mass loss at the beginning but after 300 cycles the mass loss for cRAC is about two times as much as the NAC^28^. A clear trend was seen that the mass loss increased with the portion of cRAs in concrete mixtures, which may cause by more water in cRAC than NAC, as that cRAs are more porous than NAs. Another reason is that the incorporation of cRAs in concrete weakened the binding between cRAs and new mortar and thus caused more spalling. However, the use of cRAs is beneficial to retain the FT DM loss. With the increase in the number of FT cycles, cRAC showed higher residual relative dynamic modulus than NAC. The higher percentage of replacement of NAs with cRAs, the better performance was observed. The differences between mass loss and mechanical properties can be explained by a hypothesis that mass loss is a surface phenomenon, but mechanical properties are controlled by the internal microstructure, so the porous network of cRAs is able to dissipate hydraulic pressure induced by frozen water^28^.

Alkali-silica reaction (ASR) is a pathology which could affect recycled concrete to an even larger extend than in the original concrete, due to the higher alkalis content of RAs. A recent study compared reactive NAs (Tournaisis siliceous limestone) with cRAs to investigate their effects on ASR^27^. The results showed that concrete samples prepared with reactive NAs have the highest measured ASR expansions regardless of temperature and cement-aggregates ratios, which might attribute to the dense microstructure of NAs so ASR can cause the expansion of aggregates, while cRAs can decrease ASR expansion due to the lower content of alkalis than reactive NAs.

Based on the above review, it is very clear that the durability of cRAC is in general lower than NAC. Pre-soaking cRAs in water may help to enhance the durability, but no matter which carbonation method is used, the durability of cRAC cannot reach the same performance as NAC. This is due to the natural weakness of RAs, particularly the very porous old mortar and weak ITZs. Note that most literature data in Table 1 were obtained from the Type A RAs which contain less than 5% impurities (e.g., bricks, wood chips, clay), so the Type B RAs with up to 30% impurities would have even lower durability performance (the classification of RAs types is based on the standard EN 206). To further enhance the durability performance of cRAC, RAs must be modified to have the similar properties to NAs, but there is no such an economic carbonation method available. It is reasonable to compare the durability performance of cRAC with RAC.

### Comparison with recycled concrete with noncarbonated RAs (RAC)

**Fig. 4:**
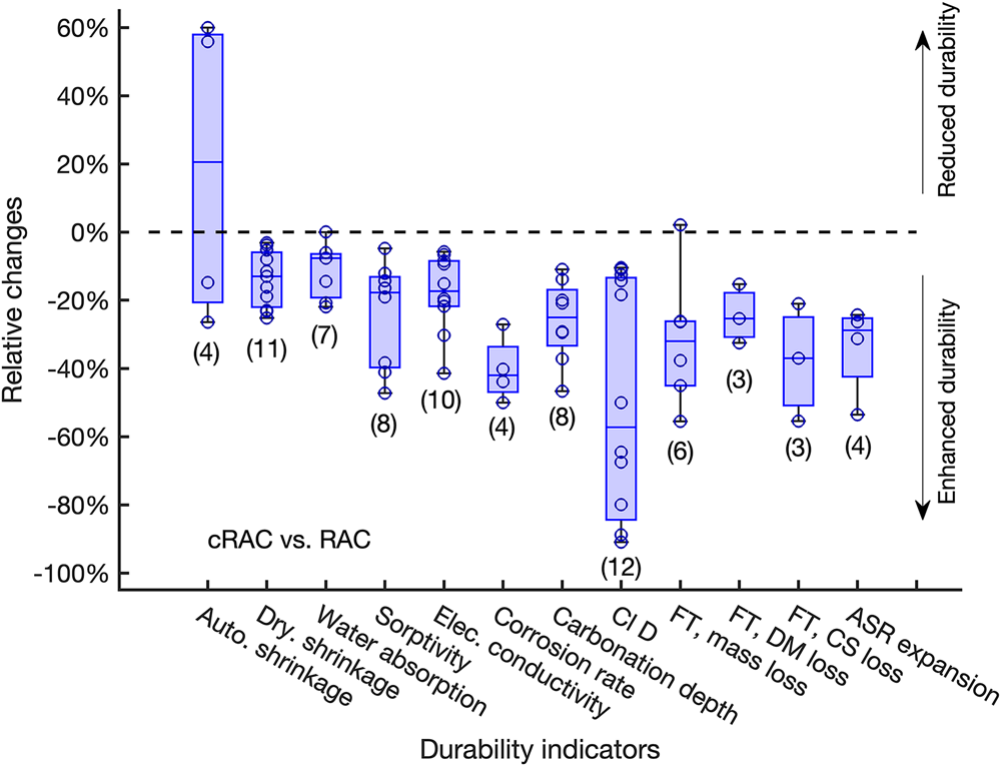
Box plots of relative changes of durability indicators of cRAC compared with RAC. This figure is plotted in the same way as Fig. 3.

As the carbonation treatment of RAs can enhance their properties, the durability of cRAC is expected to be enhanced compared with RAC. The compared durability indicators are shown in Fig. 4. Most relative values of durability indicators are negative, meaning that the durability performance of cRAC is better than RAC.

Contradictory results are found for measured autogenous shrinkage, where pre-soaking is the key to control whether autogenous shrinkage is increased (without pre-soaking^20^) or reduced (with pre-soaking^51^). The reduction in drying shrinkage due to the carbonation treatment of RAs decreases with time and the most significant changes happened in the first week of tests^20^. The higher degrees of carbonation may lead to the more reduction in drying shrinkage, such as carbonation with high CO_2_ concentration and longer duration. This might be due to more formed calcium carbonate so the water-accessible porosity of cRAC was reduced more. The reduction in drying shrinkage has a good correlation with the decrease in water absorption and sorptivity, which are all around 10%^31,32,48,52^. The reduced sorptivity indicates a higher resistance of cRAC against water penetration and the lower capillary pore volume in cRAC than RAC. The reduction rate in sorptivity is clearly related to the carbonation duration, which was about 9% reduction for 1-d carbonation but 26% reduction for 7-d carbonation^48^.

The reduction in transport properties was also reported for electrical conductivity and chloride diffusion coefficient. The measured electrical conductivity was reduced by 19% in average^11,32,48^, which had a very good correlation with sorptivity. The reduction in chloride diffusion coefficient was found to be much more significant than other transport coefficients, about 51% in average^25,31,32,48,56^, and even 80–90% of reduction was reported in some studies^20^. The reasons of such big differences are not clear, but it is most likely caused by the densification by calcium carbonate precipitation in attached old mortar after carbonation treatment^31^. As the water-accessible porosity was reduced after using cRAs, CO_2_ diffusion was slower, resulting in slow carbonation of concrete and shallow carbonation depth^32,57^. The reduction in carbonation depth seemed to be more pronounced for the longer carbonation^32^. The measured corrosion rate induced by chloride ingress was reduced by about 40% as shown in Fig. 4^25,58^. The corrosion initiation was delayed if cRAs were used because ITZs in cRAs are enhanced by carbonation so chloride ingress is slowed down and less ions can reach the steel-concrete interface^58^.

The incorporation of RAs showed both positive and negative effects on concrete samples subjected to FT cycles. The positive effect was from the more permeable aggregates that easily released the hydraulic pressures induced by the volumetric expansion of the frozen water. The negative effect was from the higher porosity of RAs than NAs, which resulted from the porous old mortar and the micro-cracks. After carbonation of RAs, the porosity was reduced and thus the FT mass loss was significantly reduced, and this reduction was more pronounced for the longer carbonation duration. This had a clear correlation with the amount of water in aggregates (porosity), as that after carbonation cRAs contained less water than RAs. With the increase in the number of FT cycles, concrete with cRAs showed the higher FT DM loss than RAs. cRAC always had the better results than RAC at the same replacement ratio^15,28^. The measured FT CS loss decreased in the sequence of RAC, cRAC, and NAC, but the best performance was found for the partial replacements of NAs with RAs (20%) or cRAs (50%)^28^. The longer carbonation of RAs, the higher residual compressive strength was seen.

The recycled concrete with cRAs showed improved resistance towards sulfate attack. Carbonation can largely reduce the amount of alkalis in reactive RAs, so the measured ASR expansion of cRAC was lower than the reactive RAs that had a high alkali content boosted by NaOH in the lab and the reduction was more pronounced for low C/A ratio^27^. Enhanced performance of cRAC in the sulfate environment was attributed to the lower water absorption and low porosity of treated RAs due to densification^15^.

The above review only considers the 100% replacement of NAs with (c)RAs. Different replacement ratios influence the durability of recycled concrete to varying degrees. According to Xuan et al.^11^, water absorption, chloride permeability, and gas permeability increase linearly with the increasing replacement ratios when compared to NAC. However, regardless of the replacement level, recycled concrete incorporating cRAs consistently shows lower values for these durability indicators than concrete with non-carbonated RAs. This suggests that using cRAs enhances the durability of recycled concrete across all replacement ratios. At low replacement levels (e.g., 20%), there is little to no impact on bulk electrical conductivity and porosity. Therefore, depending on the intended application and exposure conditions, low replacement ratios of NAs with (c)RAs are generally acceptable. For instance, the Swiss standard SIA 2030-2021 permits the use of 25% replacement (and up to 50% with prior testing) for all exposure classes related to carbonation-induced corrosion^59^.

In summary, most measured results in the literature for the durability indicators showed that carbonation treatment of RAs can obviously improve the durability of recycled concrete. The key affecting factor is that carbonation treatment of RAs reduces the water-accessible porosity and densifies the ITZs between old mortar and aggregates.

### Overview of durability performance of cRAC

To compare the overall durability performance of concrete with different types of aggregates, we calculated the ratios of measured indicators for RAC or cRAC to that for NAC in the same study, by using the equation

**Fig. 5:**
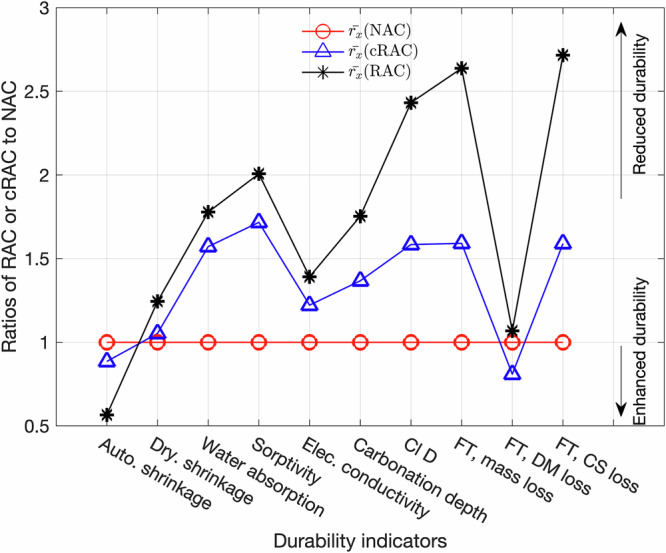
Comparison of durability indicators amongst NAC, RAC and cRAC. Indicators with high values mean worse durability performance than NAC.

${r}_{x}({\rm{RAC\; or\; cRAC}})=\frac{{y}^{x}\left({\rm{RAC\; or\; cRAC}}\right)}{{y}^{x}\left({\rm{NAC}}\right)}$. The calculated ratios from different studies were then averaged to obtain $\bar{{r}_{x}}({\rm{RAC\; or\; cRAC}})$ and the results for compared durability indicators are plotted in Fig. 5. For NAC, $\bar{{r}_{x}}=1$, while for RAC or cRAC, if an indicator is higher than 1, it performs better than NAC, and the lower indicator means the worse performance. The general trend is very clear: most durability indicators (except for autogenous shrinkage and FT dynamic modulus loss) show RAC > cRAC > NAC. The large difference in autogenous shrinkage is due to pre-soaking (which will be discussed later). The better performance of cRAC in FT dynamic modulus loss was explained by the fact that the porous microstructure can dissipate the pressure created by water freezing^28^. A careful look at Fig. 5 reveals that for most durability indicators carbonating the recycled aggregates leads to an improvement of durability performance. However, Fig. 5 suggests that the quality of cRAs need to be further improved to reach the similar durability performance to NAC.

## Discussion and research suggestions

### Improved RAs carbonation methods

The above review suggests that direct carbonation of RAs can to some extent enhance the durability performance of cRAC, but most durability indicators are still lower than NAC. Therefore, the current carbonation strategies need to be optimized to further improve the durability of cRAC.

#### Enhancing the cleanness of RAs

Enhancing the cleanness of RAs is the most straightforward way, such as minimizing the amount of attached old mortar. Different old mortar removal techniques have been studied and in general the use of high concentration acid achieved a higher removal rate than mechanical and thermal methods. As shown in Table 2, pre-soaking in 1 M HCl acid for 24 h is able to remove about 93% old mortar, resulting only about 2% old mortar attached on the original aggregates^60^. The ball milling method or the use of abrasion machine is also very efficiency, with above 80% removal rate. In comparison, the mix & wash method or heating RAs to 500 °C has a lower removal rate. At the same concentration, H_2_SO_4_ acid has a better performance than HCl and HNO_3_ acids^61^, but the high acidic concentration has a risk to react with carbonates in the original aggregates. The efficiency of thermal treatments depends on the applied temperature. The higher temperature, the larger mass loss of RAs is observed^62^, but when the temperature is above 750 °C, calcite can be partially decomposed. The efficiency also depends on the particle size of RAs. Old mortar on the smaller size of RAs is easier to be removed by either acids^63,64^ or mechanical milling^14^ because of their high surface areas. The old mortar removal rate has a direct effect the properties of the recycled concrete. After removing old mortar by HCl, the compressive strength of recycled concrete can reach 91–98% of that of NAC^60^. After the ball milling treatment to RAs, the water absorption rate of recycled concrete is increased by about 11% compared to NAs concrete, while it is 116% compared to nontreated RAs concrete^14^.

**Table 2:** The old mortar removal rate by different techniques

Clean techniques	Ball milling^14^	Abrasion machine^60^	Mix & wash^91^	HCl, 0.5 M^63^	HCl, 1 M^60^	Heating at 500 °C^60^
Mortar removal rate	82%	83%	63%	91%	93%	62%

However, as can be seen in Table 2, the removal rate of acids is already very high, but properties of recycled concrete are still not as good as NAC. To further elevate the removal efficiency, the amount of energy and cost will significantly increase for this last few percent. Additionally, the removed old mortar by acids is heavily contaminated with chemicals and cannot be directly used to prepare concrete, so another waste material is generated^21^. A high removal rate also means more recycled concrete fines, which are difficult to be reused in recycled concrete because of the variations in chemicals and physical geometries and the high cost^65^. Based on these considerations, it is not economic and environmentally friendly to continuously enhance the removal rate. The more realistic way is to increase the carbonation efficiency and the quality of cRAs.

#### Carbonation optimization and properties enhancement

##### Effect of moisture conditions

The initial moisture condition of RAs influences carbonation process and carbonation depth for a given time and CO_2_ concentration. For RAs initially with a high moisture content, liquid water can significantly slow down CO_2_ penetration and CaCO_3_ mainly participates in the region close to concrete surface. The carbonated surface layers of recycled aggregate will become a denser shield that obstructs CO2 entry^66,67^ so it would be more difficult for the reaction to occur in the deeper region. For dry RAs, at the beginning of carbonation, CO_2_ can diffuse deeper into the old mortar as the empty pore space is available. If the increase of moisture content co-exists with CO_2_ diffusion, it is expected that carbonation can happen far inside the old mortar. Therefore, the conventional carbonation methods with the constant moisture condition needs to be improved. To accelerate carbonation, the cyclically changed CO_2_ concentration and moisture condition in the chamber could help CaCO_3_ precipitate deeper and more uniform inside old mortar. The ideal cyclic moisture condition should let CO_2_ diffuse into the pore structure at low RH and let enough moisture enter the material at high RH but lower than the saturated condition, so the direct contact with liquid water needs to be avoided. This moisture condition can be addressed by utilizing emerging RH control technologies, such as atomized water droplet technology, which generates micro size water droplets rather than the bulk liquid water to moisten the carbonation environment and can be applied to concrete carbonation at both laboratory and industrial scales.

**Fig. 6:**
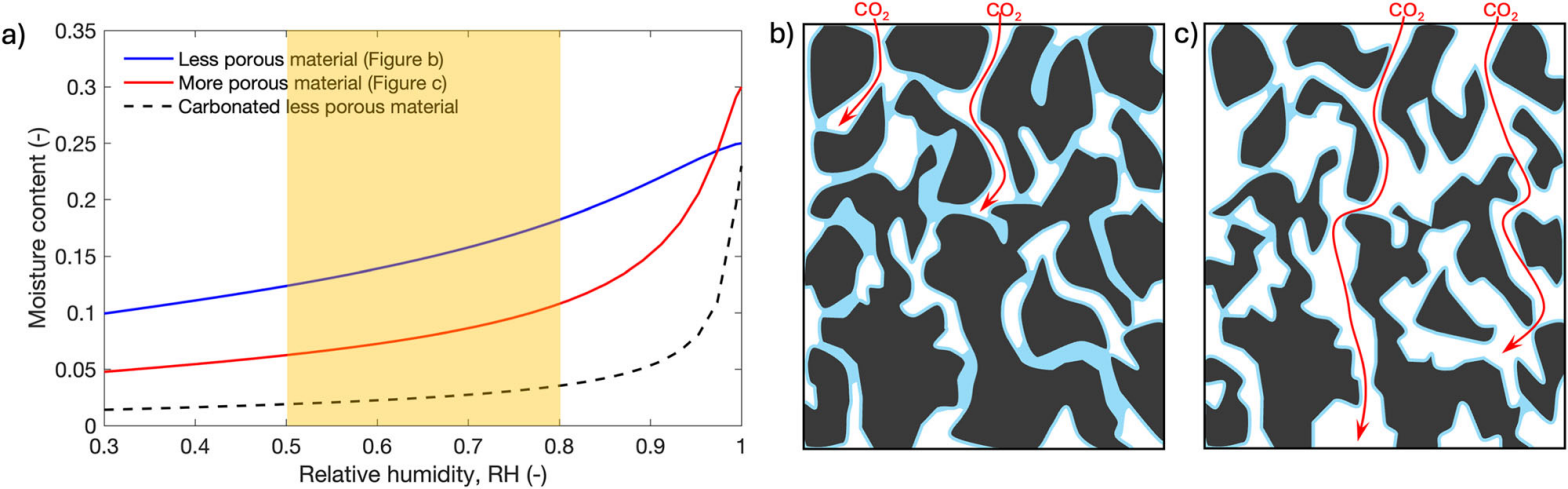
Schematic illustration of water content in different porous materials: **a** water vapor sorption isotherms for different types of materials; **b**, **c** water (blue) distribution in the less porous (**b**) and the more porous (**c**) materials at the same RH, in which the red curves represent the possible paths for CO_2_ penetration.

Before carbonation, RAs should be preconditioned at the same RH within a carbonation chamber, so there will be limited moisture transport during carbonation. The optimal RH for carbonation may depend on the microstructure and the moisture retention property of the old mortar attached on RAs. As illustrated in Fig. 6a, the water retention curves for more porous and less porous materials are very different. At the optimal RH (between 50 and 80% adapted by many studies^15,27,28^) for carbonation, the more porous material may not be able to retain the same amount water as the less porous material, because it has fewer small pores that can be filled with water in this RH range^68^. Consequently, water in the less porous material may block the pathway for CO_2_ diffusion, while at the same RH, the pathway in the more porous material for CO_2_ diffusion is well connected (see illustration in Fig. 6b, c) but the amount of water may not be the optimal for carbonation reactions. The microstructural properties and the moisture retention capacity of the old mortar attached on RAs are generally unknown, but it is commonly agreed that the old mortar on RAs is more porous than the intact mortar as discussed in Section “Effects of carbonation on microstructural properties of RAs”. Therefore, carbonation at high RH is expected to have a deeper CO_2_ penetration.

##### Enhancement of CO_2_ diffusion

Carbonation rate is generally high at the beginning and reduces with the decrease of porosity. By adjusting CO_2_ concentration, CO_2_ pressure and environmental humidity, the carbonation rate can be accelerated. Additionally, other methods were also reported to enhance the diffusion of gas or liquid in cementitious materials, such as incorporating with nano-cellulous fibers (NCFs) and sisal fibers. The presence of NCFs can change the diffusion of water through the hydration products, which is called short-circuit diffusion (SCD)^69^. Water molecules can diffuse into the inner particle with the aid of NCFs as the transport channels. NCFs can change the carbonation process and the morphology of carbonation products^70^, but it is not clear if it can enhance CO_2_ diffusion. Nevertheless, the effect of SCD may help the diffusion of calcium ions. More efficient CO_2_ penetration through the hollow lumens in sisal fibers was found^71^. The experimental results showed that both the carbonation depth and the compressive strength of composites were multiplied by the sisal fibers. Therefore, hollow natural fibers can be used as a new approach to improve the CO_2_ sequestration and mechanical performance of cementitious composites. To apply them, RAs need to be soaked in water with fibers, so fibers can remain in large pores and cracks in RAs, which will then serve as the diffusion channels for CO_2_.

##### Increasing CaCO_3_ precipitation rate

The precipitation rate of CaCO_3_ in concrete is controlled by different factors. Except moisture condition and CO_2_ diffusion, studies found that the dissolution of CO_2_ into pore water is one of the most important limiting factors^72^. To accelerate CO_2_ dissolution (or increase the concentration of HCO_3_^−^), bacteria have been added into fresh concrete and enhanced carbonation was reported^40,41^. This function of bacteria is mainly from a group of enzymes called carbonic anhydrase. Studies showed that carbonic anhydrase can enhance CO_2_ dissolution in water by thousands to millions of times^73^. By adding carbonic anhydrase into fresh cement paste, the carbonation rate is accelerated in a certain period^74^. The cracked mortar can be healed after adding carbonic anhydrase solution in the crack and then exposed to accelerated carbonation^75^. If pre-soaking RAs with carbonic anhydrase solution, the carbonation rate could be largely increased during the carbonation treatment process.

##### Manipulating CaCO_3_ precipitation location

The effect of carbonation on the microstructure of RAs depends on the location of CaCO_3_ precipitation. Conventionally, this aspect is rarely discussed in the literature. The location of CaCO_3_ precipitation depends on several factors, such as moisture condition, CO_2_ concentration, and temperature. The solid CaCO_3_ is formed at the location where sufficient amounts of CO_3_^2−^ and Ca^2+^ ions meet. Generally, this is at the gas–liquid interface, but on a microscopic scale, this location can be manipulated by changing carbonation conditions. At low moisture content, liquid water only exists in small pores, so CaCO_3_ precipitation can block these small pores. At high moisture content, the gas-liquid interface is in the large pores and CaCO_3_ precipitation cannot fill the large pores in a short time. However, if CO_2_ concentration is high, Ca^2+^ ion at the interface is consumed quickly, which leads to the retreat of CaCO_3_ precipitation to the small pores. If the temperature is high, CO_2_ dissolution is low, and the concentration of Ca^2+^ is high; thus, CaCO_3_ precipitation prefers at the gas-liquid interface. The water-filled pores may be blocked by precipitated CaCO_3_. Therefore, by adjusting the carbonation conditions, the location of CaCO_3_ precipitation can be manipulated to achieve the optimized microstructural alteration for the durability enhancement.

##### Modification of carbonates morphology

The properties of carbonated RAs also depend on the polymorphs of CaCO_3_. The stable polymorph-calcite—exhibits small cubes and are densely compact, while aragonite crystals have a long rod like or needle like morphology, which require more space to grow and increase the roughness of material surface. Taking the advantage of this morphology, the mechanical interlocking effect is enhanced if applying coating to the sample surface^76^. A recent study modified the surface morphology by adding magnesium before carbonation and then a complex phase of magnesium-calcium carbonate is formed which show hydrophobic properties^77^. Therefore, by changing the carbonation condition and the chemical composition, the carbonate products may have different properties and morphologies which could be beneficial for enhancing properties of cRAC. Nevertheless, one of the main problems is the long-term stability of aragonite is unpredictable under the service condition, so further studies should focus on methods to stabilize the meta stable carbonate polymorphs.

### Effect of aggregate moisture content

Experimental data in the literature showed that RAs with different initial moisture contents have various effects the durability of recycled concrete. Saturated RAs can reduce autogenous shrinkage of recycled concrete compared to oven-dry or natural state RAs because saturated RAs can supply sufficient water for internal curing^78^. This is also used to explain the reduced drying shrinkage of concrete with saturated RAs. Regarding other durability indicators (drying shrinkage^79,80^, FT resistance^81^, and chloride ingress^80,82^), the effects of initial moisture contents in RAs on concrete durability vary. Zhao et al.^80^, reported that RAC with natural state RAs has a better performance on chloride ingress but low performance of drying shrinkage than that with pre-wetted RAs. However, another study showed that the durability indicators (water absorption coefficient, electrical conductivity and chloride D) are lower for RAC with moderate moisture content RAs (50–65% of saturation) but higher for that with natural state RAs and much higher for pre-wetted RAs^82^. As for FT resistance, the better results for semi-saturated RAs (~88% saturation) than the saturated and dry RAs were observed^81^. This may be due to the higher ITZ quality if the partially saturated RAs are used in recycled concrete^81^, which was later confirmed by the measured microhardness of ITZ, showing the unsaturated RAs have the higher microhardness of the ITZ than pre-wetted RAs, even higher than that of NAs^82^. Although no such comparisons for cRAs are available in the literature, we anticipate that the effect of moisture content in cRAs may be similar or less pronounced than RAs as the water-accessible porosity of cRAs is lower than RAs.

### Contradiction in moisture absorption

With pre-soaking in water, autogenous shrinkage of cRAC is lower than that of RAC, while without pre-soaking in water, autogenous shrinkage of cRAC is significantly higher. This implies that cRAs absorb more water from fresh concrete than RAs, which is also observed in many engineering practices and unpublished data. This observation seems to contradict with the fact that the porosity of cRAs is lower than RAs so it is not expected that cRAs can absorb more water than RAs. However, we believe this may be due to the different moisture retention capacities of RAs and cRAs. It is commonly found that after carbonatation the pore structure of a cementitious material is coarsened^83,84^, so if these aggregates are stored at the same RH (e.g., the lab RH, about 50%), moisture content in cRAs is lower than the non-carbonated RAs as shown by the dashed-black sorption isotherm illustrated in Fig. 6a. After these aggregates are mixed in cement slurry, they absorb water to reach a very high moisture content. Even though the porosity of cRAs is lower than RAs, they can take more water from cement slurry. Therefore, pre-soaking or pre-wetting of RAs is a good practice to mitigate water absorption from cement slurry and thus reduce the shrinkage at the early ages.

### Potential risk

The aims of carbonation of RAs are to (1) enhance the properties of RAs, and (2) maximize CO_2_ uptake. The ideal carbonation is that portlandite (CH) is fully converted to CaCO_3_ and a very low amount of C-S-H and ettringite is carbonated. However, due to the uneven carbonation rate between the surface layer and the inner part of an RA and different efficiencies of various carbonation techniques, the old mortar at the surface of an RA may be over carbonated, leading to C-S-H and ettringite being heavily carbonated. The products of carbonation of C-S-H are CaCO_3_, silica gel and water^85,86^, and the carbonation of ettringite leads to as much as 50% volumetric shrinkage^87^, so over carbonation may cause an increase in porosity and a reduction of mechanical properties of old mortar. Even though over carbonation will lead to a higher CO_2_ uptake by RAs, the over carbonated RAs can impair the durability of cRAC. Therefore, when using the carbonation treatment of RAs, the carbonation condition and duration need to be well controlled to avoid over carbonation but keep the balance between properties enhancement and CO_2_ uptake.

Even though the research results showed that the carbonation treatment of RAs can mitigate ASR expansion compared with reactive NAs, no studies focused on the comparison with non-reactive NAs are available in the literature, which are expected to have much lower ASR expansion than reactive NAs. If a certain amount alkali remains, the risk ASR is still possible. Nevertheless, because of the porous nature of cRAs, the expansion pressure created by ASR may be dissipated. Such effects need further studies to reveal.

## Conclusion

This paper reviews the durability performance of cRAC. Compared with NAC and RAC, the following conclusions can be drawn.

- Most durability indicators (shrinkage, transport properties, FT resistance) of cRAC are lower than NAC, because the quality of cRAs is not as good as NAs.
- Durability of cRAC is significantly improved compared with RAC as the microstructure of RAs is densified, and alkalinity is reduced after carbonation. While the overall durability performance of cRAC was between NAC and RAC, there are still potential methods to further improve the quality of cRAs.
- The initial moisture content of aggregates is one of the key affecting factors on durability of recycled concrete. The water-accessible porosity of cRAs is higher than NAs, so cRAs need more water to have a sufficient carbonation which can further enhance the durability of cRAC. However, it is not clear which initial moisture content is optimal for concrete durability, so more studies are still needed.

To enhance the quality of cRAs, we suggest that: (1) Carbonation methods can be optimized to further improve the durability performance of cRAs by adjusting moisture content, temperature, CO_2_ concentration, and carbonation duration depending on the microstructural properties of RAs; (2) To enhance carbonation efficiency, CO_2_ diffusion and CaCO_3_ precipitation need to be accelerated by incorporating bio- or nano-materials, and (3) The over carbonation should be avoided.

## Data Availability

The collected data shown in Figure 3, Figure 4, and Figure 5 are available upon request.
